# Epicardial Adipose Tissue as a Cardiometabolic Target in Atrial Fibrillation: Implications for Ablation Strategies and Emerging Metabolic Therapies

**DOI:** 10.3390/medsci14010127

**Published:** 2026-03-09

**Authors:** Fulvio Cacciapuoti

**Affiliations:** Division of Cardiology, “Antonio Cardarelli” Hospital, 80131 Naples, Italy; fulvio.cacciapuoti@aocardarelli.it; Tel.: +39-081-7474011

**Keywords:** epicardial adipose tissue, atrial fibrillation, echocardiography, catheter ablation, GLP-1 receptor agonists, GIP/GLP-1 dual agonists

## Abstract

Background: Atrial fibrillation (AF) is a prevalent arrhythmia closely associated with cardiometabolic disorders and systemic inflammation. Epicardial adipose tissue (EAT), located in direct contact with the atrial myocardium, has emerged as a biologically active tissue involved in atrial remodeling through inflammatory, fibrotic, and electrophysiological mechanisms. The objective of this review is to summarize current translational and clinical evidence on the role of EAT in AF pathophysiology and to discuss its implications for diagnostic assessment, interventional management, and cardiometabolic therapeutic strategies. Methods: A narrative, structured review of experimental, translational, and clinical studies was conducted using major biomedical databases. The literature was evaluated with a focus on mechanisms linking EAT to atrial remodeling, noninvasive imaging techniques for EAT characterization, echocardiographic and electroanatomical markers of atrial disease, outcomes of catheter ablation strategies, and pharmacological interventions targeting metabolic and inflammatory pathways. Results: The available evidence indicates that increased EAT volume and altered inflammatory activity are associated with atrial fibrosis, conduction abnormalities, and impaired atrial function, contributing to AF initiation and persistence. Multimodality imaging, including cardiac computed tomography and cardiac magnetic resonance, enables quantitative and qualitative assessment of EAT and supports clinical phenotyping. Clinical studies report an association between higher EAT burden and increased AF recurrence after pulmonary vein isolation, particularly in patients with persistent AF. Emerging cardiometabolic therapies, such as glucagon-like peptide-1 receptor agonists and dual GIP/GLP-1 agonists, have been shown to reduce EAT volume and inflammatory markers, although direct evidence linking these interventions to improved AF outcomes remains limited. Conclusions: EAT represents a relevant pathophysiological interface between metabolic disease and AF with potential clinical implications. Incorporating EAT assessment into routine evaluation may enhance risk stratification and support personalized AF management. Further prospective studies are required to define its role as a therapeutic target in clinical practice.

## 1. Introduction

Epicardial adipose tissue (EAT) is a specialized visceral fat depot located between the myocardium and the visceral layer of the pericardium [1]. It is uniquely positioned in close proximity to the cardiac muscle and shares its microcirculation, which facilitates direct paracrine and vasocrine interactions with the underlying myocardium [2]. Under physiological conditions, EAT performs several beneficial functions, including mechanical protection of coronary arteries, thermoregulation, and secretion of anti-inflammatory adipokines [3]. However, in pathologic states such as obesity, insulin resistance, and systemic inflammation, EAT undergoes phenotypic changes characterized by hypertrophy, inflammatory cell infiltration, and a shift toward a pro-inflammatory and profibrotic secretome [4].

Mounting evidence implicates EAT in the development and progression of atrial fibrillation (AF) [5]. Through the release of cytokines (e.g., interleukin-6, TNF-α), chemokines, and extracellular matrix-modulating enzymes, EAT promotes structural remodeling of the atrial myocardium, including fibrosis and adipocyte infiltration [6]. These changes disrupt the normal atrial architecture and contribute to conduction heterogeneity, delayed atrial conduction, and increased susceptibility to arrhythmogenesis [7]. In this context, EAT functions not merely as a passive bystander but as an active contributor to the arrhythmic substrate.

Echocardiographic and advanced imaging studies have demonstrated that increased EAT volume correlates with impaired atrial function, reduced atrial strain, and prolonged atrial conduction time—all predictors of AF onset and recurrence [8]. Moreover, EAT burden appears to negatively influence the efficacy of rhythm control strategies such as catheter ablation [9].

Given the growing prevalence of obesity and AF, understanding the mechanistic role of EAT is essential for identifying novel therapeutic targets. This review aims to explore the pathophysiological mechanisms linking EAT to AF, the diagnostic and prognostic value of imaging-derived EAT metrics, and emerging therapeutic strategies—including catheter ablation approaches and novel metabolic agents such as GLP-1 and GIP receptor agonists—that may modulate EAT and improve AF outcomes [10].

In this narrative review, a literature search was conducted in PubMed/Medline and Scopus to identify experimental, translational, and clinical studies investigating the relationship between EAT and AF. The search focused on the following domains: (i) mechanistic links between EAT and atrial remodeling (inflammation, fibrosis, electrophysiology), (ii) EAT assessment by echocardiography, cardiac CT, and cardiac magnetic resonance, (iii) associations between EAT and electroanatomical mapping features and ablation outcomes, and (iv) cardiometabolic interventions potentially modulating EAT, including weight loss strategies and incretin-based therapies (GLP-1 receptor agonists and dual GIP/GLP-1 agonists). Priority was given to peer-reviewed studies in humans; selected experimental studies were included when providing mechanistic support. Reference lists of key articles were also screened to identify additional relevant publications.

## 2. Distribution, Physiological Role, and Contribution to Atrial Fibrosis of Epicardial Adipose Tissue

**Figure 1:**
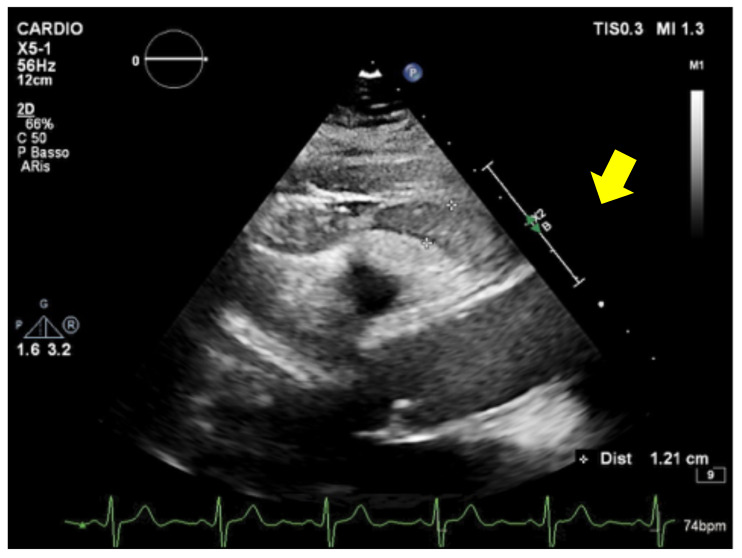
Transthoracic echocardiography showing epicardial adipose tissue. Parasternal short-axis view shows an echolucent layer consistent with epicardial adipose tissue (arrow).

EAT is predominantly distributed along the atrioventricular and interventricular grooves, surrounding the coronary arteries, and enveloping both atria—particularly the posterior left atrial wall and the left atrial appendage [11]. This fat depot is unique in that it lies directly over the myocardium, with no separating fascia, and shares a common embryologic origin with visceral fat (Figure 1).

Under normal conditions, EAT exerts cardioprotective functions, including mechanical cushioning of coronary vessels, fatty acid storage and supply to the myocardium, thermoregulation, and secretion of anti-inflammatory adipokines such as adiponectin [12].

However, in pathological states such as obesity, type 2 diabetes, and metabolic syndrome, EAT undergoes expansion and phenotypic transformation [13]. The resulting tissue exhibits increased infiltration of inflammatory cells (e.g., macrophages, T lymphocytes) and shifts its secretory profile toward pro-inflammatory cytokines (e.g., IL-6, TNF-α), profibrotic mediators (e.g., TGF-β), and matrix metalloproteinases [14]. These molecules diffuse locally to the adjacent atrial myocardium, where they promote oxidative stress, myocyte apoptosis, and fibroblast activation [15]. This paracrine signaling fosters interstitial fibrosis, alters atrial conduction velocity, and increases electrical heterogeneity, all of which contribute to the substrate for AF [16]. Notably, fibrotic remodeling tends to be most pronounced in regions with high EAT density, such as the posterior left atrial wall, which may explain the increased arrhythmogenicity of these areas and their relevance in ablation strategies [17].

Key clinical and imaging studies supporting the link between epicardial adipose tissue, atrial structural and electrophysiological remodeling, and atrial fibrillation are summarized in Table 1.

## 3. Cardiac CT and MRI in the Assessment of Epicardial Adipose Tissue

**Figure 2:**
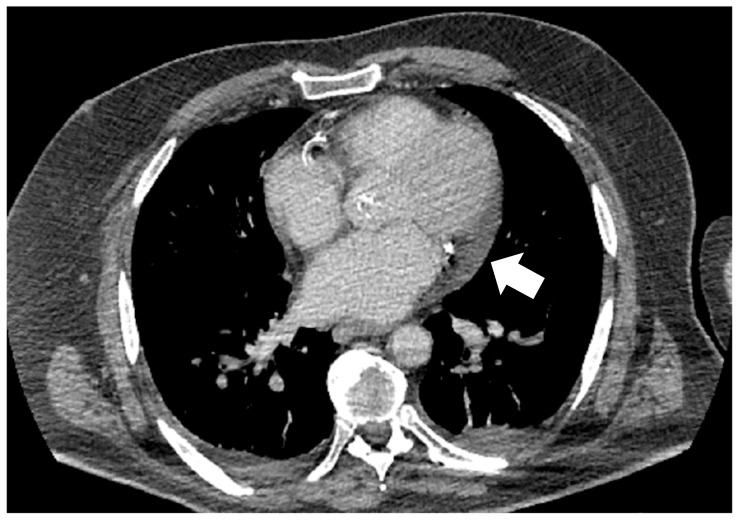
Cardiac computed tomography illustrating periatrial epicardial adipose tissue (arrow).

Cardiac imaging modalities such as computed tomography (CT—Figure 2) and magnetic resonance imaging (MRI) provide detailed, reproducible quantification of EAT and play an increasingly important role in risk stratification and procedural planning in AF [21]. Non-contrast ECG-gated cardiac CT is considered the gold standard for EAT volume assessment due to its high spatial resolution and ability to distinguish fat based on its characteristic attenuation (typically −190 to −30 Hounsfield units) [22]. CT allows for precise quantification of EAT volume and distribution, particularly around the left atrium and pulmonary veins, where fat accumulation is most relevant to arrhythmogenesis [23]. In addition, periatrial EAT thickness measured on CT has been correlated with low-voltage zones and fibrotic remodeling seen on electroanatomical mapping [24].

Cardiac magnetic resonance imaging (CMR) offers complementary advantages, including superior tissue characterization without ionizing radiation [25]. While CMR-based fat quantification is less commonly used in clinical practice, advanced techniques such as Dixon sequences or fat–water separation imaging enable reliable estimation of EAT volume and characterization of adjacent myocardial tissue [26]. Importantly, CMR also permits late gadolinium enhancement (LGE) imaging to detect atrial fibrosis, which can be spatially correlated with epicardial fat distribution—highlighting the mechanistic link between EAT and atrial remodeling [27]. Moreover, combining EAT volume measurements with fibrosis burden on CMR may improve the prediction of AF recurrence after catheter ablation.

Both CT and CMR thus provide valuable anatomic and functional data that enhance the understanding of EAT’s role in AF and support a phenotype-driven approach to patient management.

## 4. Echocardiographic Assessment of Atrial Function in Patients with Increased Epicardial Adipose Tissue

**Figure 3:**
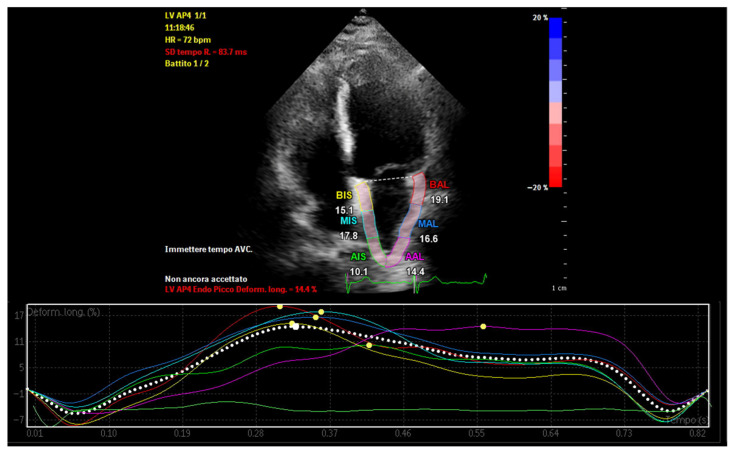
Speckle-tracking echocardiography illustrating left atrial functional impairment in the presence of increased epicardial adipose tissue. Reduced left atrial reservoir strain and segmental dyssynchrony reflect early atrial mechanical remodeling associated with epicardial fat accumulation.

In patients with abundant EAT, echocardiographic evaluation reveals distinct alterations in atrial mechanics that reflect early structural and electrical remodeling [28]. Speckle-tracking echocardiography (STE) has emerged as a sensitive modality for assessing atrial function through myocardial deformation parameters, particularly atrial strain (Figure 3). Left atrial strain is typically evaluated during three phases: reservoir (LASr), conduit (LAScd), and contraction (LASct). In individuals with increased EAT, a consistent reduction in LASr and LAScd is observed, indicating impaired atrial compliance and passive emptying due to atrial stiffness and interstitial fibrosis [20]. The contractile strain (LASct) may also be reduced, particularly in advanced remodeling, reflecting impaired booster pump function.

**Figure 4:**
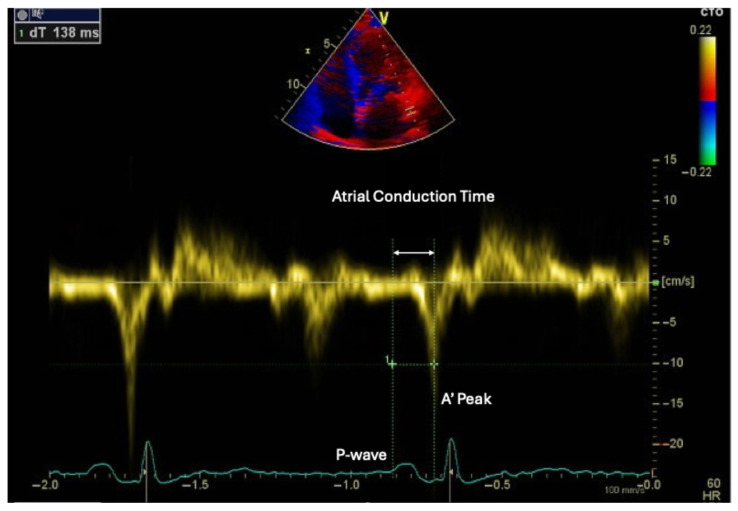
Tissue Doppler imaging assessment of atrial conduction time in a patient with increased epicardial adipose tissue. Atrial conduction time (PA-TDI) is measured from the onset of the P wave on the electrocardiogram to the peak A′ velocity at the lateral mitral annulus, reflecting delayed atrial electrical activation associated with atrial remodeling.

In addition to strain parameters, atrial electromechanical delay—typically assessed by tissue Doppler imaging (TDI) as the time interval from the onset of the P wave on ECG to the peak A′ velocity at the lateral mitral annulus (PA-TDI)—is often prolonged in patients with high EAT volume (Figure 4) [29]. This reflects slowed atrial conduction and is associated with heterogeneous and anisotropic propagation of electrical impulses due to fibrosis and fatty infiltration [30].

Furthermore, indices of atrial dyssynchrony, such as intra-atrial and inter-atrial electromechanical delay (measured as time differences in PA-TDI between septal and lateral or left and right atrial sites), are frequently abnormal in this population. Atrial mechanical dyssynchrony is thought to result from regional variability in fibrotic remodeling and inflammatory infiltration, particularly in regions where EAT is most abundant, such as the posterior and inferior left atrial walls.

Collectively, these echocardiographic findings support the concept that EAT is not merely an inert fat depot but an active driver of atrial dysfunction. The alterations in strain, conduction time, and mechanical synchrony observed in EAT-rich patients provide valuable insights into early atrial cardiomyopathy and may help refine risk stratification and timing of intervention in atrial fibrillation management [31].

## 5. Electroanatomical Mapping of the Left Atrium

In patients with paroxysmal atrial fibrillation (PAF) and increased EAT, electroanatomical mapping (EAM) of the left atrium frequently reveals early yet distinct structural and electrical alterations that suggest an arrhythmogenic substrate beyond pulmonary vein (PV) triggers [18]. High-resolution voltage mapping performed during sinus rhythm typically shows areas of low-voltage (<0.5 mV) or fractionated electrograms in the posterior, inferior, and lateral walls of the LA—regions commonly adjacent to epicardial fat depots. While PAF is classically associated with preserved global atrial voltage, in patients with increased EAT, a patchy low-voltage substrate may be evident even in the absence of persistent AF or overt structural heart disease [19].

These low-voltage zones (LVZs) are thought to reflect localized fibrosis, adipocyte infiltration, and interstitial inflammation driven by paracrine effects of EAT [32]. Fractionated or prolonged electrograms in these areas are indicative of conduction slowing and heterogeneity, promoting reentrant circuit formation. Studies have demonstrated a spatial correlation between high EAT thickness on imaging (e.g., CT or MRI) and low-voltage areas on mapping, supporting the hypothesis that EAT contributes directly to arrhythmogenic remodeling [33].

Activation mapping may further reveal areas of delayed or fragmented conduction, often in the posterior wall, where epicardial fat accumulation is most pronounced [34]. The presence of such conduction abnormalities despite a paroxysmal AF phenotype suggests an early stage of atrial cardiomyopathy, sometimes referred to as “fibro-fatty atrial myopathy.” In this context, tailored ablation strategies beyond pulmonary vein isolation (PVI)—such as posterior wall isolation or ablation of low-voltage areas—may be beneficial, although data are still evolving [35].

Importantly, the identification of EAT-related atrial remodeling during EAM may inform procedural planning and prognosis, as patients with more extensive EAT and associated electrical abnormalities may have a higher risk of AF recurrence post-ablation, even in the paroxysmal stage.

## 6. Posterior Wall Electroanatomical Ablation vs. Cryoballoon PVI

In patients with PAF and high EAT burden, the posterior wall of the left atrium emerges as a key arrhythmogenic substrate [36]. This region, heavily influenced by adjacent EAT, demonstrates early structural remodeling, including fibro-fatty infiltration, localized inflammation, and conduction delay [37]. While PVI remains the foundation of catheter ablation, emerging evidence suggests that PVI alone—especially when performed using cryoballoon technology—may be insufficient in this specific phenotype, as it fails to address non-pulmonary vein triggers and diseased atrial myocardium located within the posterior wall [38]. Key studies assessing the impact of epicardial adipose tissue on catheter ablation strategies and post-procedural atrial fibrillation recurrence are summarized in Table 2.

Point-by-point radiofrequency (RF) ablation enables detailed electroanatomical mapping and precise lesion delivery, allowing not only for PVI but also for posterior wall isolation (PWI) through linear ablation (e.g., roof and inferior lines) and substrate modification targeting low-voltage and fractionated regions [40]. In contrast, cryoballoon PVI, although effective in standard PAF, offers limited substrate adaptability and does not routinely achieve posterior wall isolation [41].

Clinical data support the superiority of posterior wall modification in selected patients. In the POBI-AF trial, adjunctive posterior wall isolation in addition to PVI resulted in significantly lower arrhythmia recurrence compared to PVI alone, particularly in patients with evidence of posterior wall low-voltage areas [42]. Similarly, observational studies have demonstrated that EAT volume correlates with the extent of posterior low-voltage substrate, and that patients with greater EAT burden benefit most from tailored ablation strategies that include the posterior wall [43]. In contrast, the CAPLA trial, which compared PVI alone to PVI plus posterior wall isolation in unselected patients, did not find a significant difference—highlighting the need for phenotypic stratification, such as EAT quantification, when selecting patients who may benefit from substrate-guided approaches [39].

Therefore, in patients with paroxysmal AF and high posterior EAT burden—whether identified by cardiac imaging (e.g., CT or MRI) or inferred from electroanatomical voltage mapping—electroanatomical ablation of the posterior wall via RF may provide superior rhythm outcomes in selected patients, pending validation in prospective phenotype-driven trials. This supports a personalized, substrate-directed approach to ablation in metabolically remodeled atria.

## 7. Therapeutic Implications of Weight Loss and Incretin-Based Therapies

Weight loss represents a cornerstone intervention for reducing EAT burden and its associated pro-inflammatory and profibrotic activity [44]. Several observational and interventional studies have demonstrated that reductions in body weight are accompanied by parallel decreases in visceral and epicardial fat depots, leading to improvements in local inflammatory signaling and atrial structural remodeling [45]. Importantly, EAT reduction appears to be proportional to the magnitude of weight loss, supporting a dose–response relationship between metabolic improvement and atrial substrate modification [46].

Beyond its effects on EAT volume, weight loss has been associated with favorable changes in atrial size, compliance, and electrical stability, which are key determinants of AF susceptibility [47]. Lifestyle-based interventions, including dietary modification and increased physical activity, as well as bariatric surgery in selected populations, have been shown to reduce AF burden and improve rhythm control, although direct evidence specifically linking weight loss–induced EAT reduction to AF outcomes remains limited [48,49].

In this context, weight loss should be considered the foundational therapeutic strategy upon which pharmacological interventions act. The beneficial effects of incretin-based therapies on EAT and atrial remodeling are likely mediated, at least in part, by their robust impact on body weight, insulin sensitivity, and systemic inflammation [50]. Consequently, lifestyle-driven weight reduction and pharmacological approaches should be viewed as complementary rather than competing strategies in the management of metabolically mediated atrial fibrillation [51].

The recognition of EAT as an active contributor to atrial remodeling and arrhythmogenesis has prompted interest in pharmacological strategies targeting adipose tissue reduction to mitigate AF risk [52]. Among these, glucagon-like peptide-1 receptor agonists (GLP-1 RAs) and dual glucose-dependent insulinotropic polypeptide/glucagon-like peptide-1 receptor agonists (GIP/GLP-1 RAs) have emerged as promising agents that extend beyond glycemic control to influence adipose tissue metabolism, inflammation, and fibrosis [53].

The effects of weight loss strategies and incretin-based therapies on epicardial adipose tissue and their potential implications for atrial fibrillation are summarized in Table 3.

GLP-1 RAs, such as liraglutide and semaglutide, have demonstrated efficacy in reducing visceral and epicardial fat volumes, likely through mechanisms including appetite suppression, weight loss, increased energy expenditure, and improved insulin sensitivity [57]. These agents may exert anti-inflammatory and anti-fibrotic effects within the myocardium and surrounding adipose depots, potentially stabilizing the arrhythmogenic substrate [54].

Dual GIP/GLP-1 RAs, such as tirzepatide, have shown superior efficacy in weight reduction and visceral fat loss compared to GLP-1 RAs alone [55]. While GIP has traditionally been associated with anabolic effects on adipose tissue, GIP seems to exert lipolytic effects under certain conditions. Specifically, GIP receptor activation has been linked to increased lipolysis in human subcutaneous adipose tissue, as evidenced by elevated glycerol release, indicating enhanced breakdown of triglycerides [58]. Furthermore, GIP receptor expression is higher in visceral adipose tissue compared to subcutaneous fat, suggesting a more pronounced effect of GIP on visceral fat depots [59].

The combined activation of GLP-1 and GIP receptors may therefore synergistically promote the reduction in EAT and visceral fat, potentially ameliorating the pro-arrhythmic substrate associated with AF [56]. Although direct evidence linking these pharmacotherapies to reduced AF incidence is still emerging, the observed reductions in EAT volume and improvements in metabolic profiles support their potential role as adjunctive therapies in AF management, particularly in obese or metabolically compromised individuals [60].

From a mechanistic perspective, the potential anti-arrhythmic effects of incretin-based therapies appear to be mediated through a combination of systemic and local pathways rather than direct electrophysiological actions. GLP-1 receptor agonists and dual GIP/GLP-1 receptor agonists exert profound effects on body weight, insulin resistance, and systemic inflammation, all of which are key drivers of atrial remodeling [61]. At the local level, reductions in epicardial adipose tissue volume and inflammatory activity may attenuate paracrine signaling toward the adjacent atrial myocardium, leading to decreased fibro-inflammatory remodeling and a more stable atrial substrate [62]. Imaging studies have consistently shown parallel reductions in visceral and epicardial fat depots following incretin-based therapy, suggesting that EAT modulation largely reflects global metabolic improvement rather than a tissue-specific pharmacological effect [63].

Despite this strong mechanistic rationale, current evidence linking incretin-based therapies to atrial fibrillation outcomes remains limited. Most available data are derived from observational analyses, post hoc imaging endpoints, or indirect associations with weight loss and cardiometabolic risk reduction. Dedicated prospective studies assessing atrial fibrillation incidence, burden, or recurrence as primary endpoints are lacking, and no randomized trials have yet demonstrated a direct causal relationship between EAT reduction induced by incretin-based therapies and improved rhythm control. Therefore, while these agents represent a promising adjunctive strategy in patients with AF and metabolic dysfunction, their role in arrhythmia prevention or treatment should currently be considered hypothesis-generating and warrants validation in phenotype-driven clinical trials.

## 8. Conclusions

EAT is now recognized not only as a passive fat depot but as an active component of the atrial microenvironment that may contribute to structural and electrical remodeling in AF.

Although the available evidence is largely observational, the consistent association between EAT burden, atrial substrate abnormalities, and post-ablation recurrence supports its potential value for clinical phenotyping and risk stratification.

Weight reduction and metabolic therapies can reduce EAT volume and inflammatory activity, but at present they should be considered complementary to catheter ablation within an integrated, patient-specific management strategy.

Prospective studies are required to determine whether targeting EAT translates into improved rhythm outcomes and to define which patient subgroups benefit most.

## Figures and Tables

**Table 1:** Key clinical studies linking epicardial adipose tissue to atrial remodeling and atrial fibrillation.

Author (Year)	Study Design	Population	EAT Assessment	Main Findings
Zghaib et al. [18] (2016)	Observational	AF patients	CT-derived periatrial EAT	Higher EAT thickness associated with low-voltage areas and electrogram fractionation
Shao et al. [19] (2022)	Cross-sectional	NVAF patients	CT	Periatrial EAT independently associated with left atrial low-voltage zones
Nabati et al. [20] (2023)	Case-control	T2DM vs controls	Echo EAT thickness	Increased EAT correlated with reduced LA reservoir strain
Ma et al. [8] (2025)	Prospective cohort	Elderly hypertensive pts	Echo	EAT thickness predicted incident AF at 2-year follow-up
Huber et al. [21] (2024)	Cohort	AF post-PVI	CT EAT dispersion	Higher EAT dispersion associated with AF recurrence

AF = Atrial Fibrillation; CT = Computed Tomography; EAT = Epicardial Adipose Tissue; NVAF = Non-Valvular Atrial Fibrillation; T2DM = Type 2 Diabetes Mellitus; LA = Left Atrial; PVI = Pulmonary Vein Isolation.

**Table 2:** Epicardial adipose tissue burden and outcomes after catheter ablation of atrial fibrillation.

Study	AF Type	Ablation Strategy	EAT Measurement	Follow-up	Main Outcome
Nakahara et al. [9] (2014)	Persistent AF	RF PVI + substrate	CT periatrial EAT	12 mo	Higher EAT associated with AF recurrence
Nakatani et al. [17] (2020)	Persistent AF	PVI vs PVI + PWI	CT	18 mo	Posterior EAT predicted benefit from PWI
Qiu et al. [34] (2025)	Mixed AF	RF PVI	CT + EAM	24 mo	Combination of EAT + LVZ predicted recurrence
CAPLA trial [39] (2023)	Persistent AF	PVI vs PVI + PWI	Not stratified	12 mo	No overall benefit of PWI in unselected patients

AF = Atrial Fibrillation; RF = Radio Frequency; PVI = Pulmonary Vein Isolation; CT = Computed Tomography; EAT = Epicardial Adipose Tissue; PWI = Posterior Wall Isolation; EAM = Electroanatomical Mapping; LVZ = Low-Voltage Zones.

**Table 3:** Effects of weight loss strategies and incretin-based therapies on epicardial adipose tissue and atrial fibrillation.

Intervention	Study Type	Effect on Weight	Effect on EAT	Evidence on AF	Notes
Lifestyle weight loss [45]	Observational	Moderate ↓	↓ EAT thickness/volume	Indirect	EAT reduction proportional to weight loss
GLP-1 RA (e.g., semaglutide) [54]	RCTs/imaging studies	Significant ↓	↓ EAT volume and inflammation	Indirect	Effects partly weight-loss mediated
Dual GIP/GLP-1 RA (tirzepatide) [55,56]	RCTs	Marked ↓	Pronounced visceral/EAT ↓	Not available	Potential synergistic metabolic effects
Bariatric surgery [48]	Cohort studies	Major ↓	Marked ↓ EAT	Indirect	Strong metabolic remodeling

EAT = Epicardial Adipose Tissue; GLP-1 RA = Glucagon-Like Peptide-1 Receptor Agonist; GIP = Glucose-dependent Insulinotropic Polypeptide; RCTs = Randomized Controlled Trials; AF = Atrial Fibrillation.

## Data Availability

Data sharing is not applicable.

## Abbreviations

The following abbreviations are used in this manuscript:

EAM	Electroanatomical mapping
EAT	Epicardial adipose tissue
GLP-1	Glucagon-like peptide-1
GIP	Glucose-dependent insulinotropic polypeptide
LAA	Left atrial appendage
LGE	Late gadolinium enhancement
PA-TDI	P-wave to A’-wave tissue Doppler imaging interval
PVI	Pulmonary vein isolation
PWI	Posterior wall isolation
VAT	Visceral adipose tissue
